# Advanced Immunoassays and Biosensing: From Design to Development

**DOI:** 10.3390/bios15120822

**Published:** 2025-12-18

**Authors:** Dongyang Li, Xu Wang, Juan Pablo Salvador

**Affiliations:** 1 College of Biosystems Engineering and Food Science, Zhejiang University, Hangzhou 310058, China; 2 Zhejiang Key Laboratory of Intelligent Sensing and Robotics for Agriculture, Zhejiang University, Hangzhou 310058, China; 3 Department of Pathology, College of Basic Medical Sciences, Shanghai Jiao Tong University School of Medicine, Shanghai 200025, China; 4 CIBER de Bioingeniería, Biomateriales Nanomedicina (CIBER-BBN), Institute for Advanced Chemistry of Catalonia (IQAC) of the Spanish Council for Scientific Research (CSIC), Jordi Girona 18-26, 08034 Barcelona, Spain; 5 Nanobiotechnology for Diagnostics Group (Nb4D), Department of Chemical and Biomolecular Nanotechnology, Institute for Advanced Chemistry of Catalonia (IQAC) of the Spanish Council for Scientific Research (CSIC), Jordi Girona 18-26, 08034 Barcelona, Spain

Over the past decade, the field of immunoassays and biosensing has undergone remarkable expansion, driven by the urgent demand for sensitive, rapid, and reliable detection technologies across biomedical, environmental, and food safety applications. Progress in antibody engineering, nanomaterials, microfluidics, and bioelectronics has greatly enhanced biosensor performance, enabling precise detection of diverse analytes—from small molecules and proteins to pathogens and viral nucleic acids. The interdisciplinary integration of immunology, chemistry, materials science, and bioengineering has further accelerated innovation, paving the way for versatile platforms in point-of-care testing, high-throughput screening, and environmental surveillance.

The aim of this Special Issue on “Immunoassays and Biosensing” is to highlight the most recent scientific and technological advances shaping this dynamic field. The Special Issue features 13 contributions, including original research, communications, and reviews, covering topics ranging from the molecular design of high-performance recognition elements to the engineering of integrated sensing devices. Collectively, these works illustrate the fundamental insights and practical applications driving the next generation of biosensing technologies.

## 1. Innovative Assay Strategies and Sensing Platforms

Several of the studies in this Special Issue focus on rapid detection technologies, particularly lateral flow immunoassays (LFIAs). Sotnikov et al. address the classic challenge associated with competitive LFIAs: balancing antibody usage with sensitivity by introducing a novel dual gold nanoparticle conjugate strategy (Contribution 1). This approach significantly broadens the working range and has been successfully applied for the ultrasensitive detection of imidacloprid in honey. In a similar vein, Xu et al. and Chen et al. developed highly sensitive and rapid LFIA methods for organophosphorus (Contribution 2) pesticides and pyridaben (Contribution 3), respectively, demonstrating the critical role of antibody engineering in enhancing on-site detection performance. Beyond conventional LFIAs, researchers are also exploring more sophisticated sensing mechanisms. Yang et al. constructed a sandwich-type electrochemical immunosensor for Tau protein detection, with a wide linear range and low background interference, thereby presenting a promising tool for clinical diagnostics of Alzheimer’s disease (Contribution 4). Chen et al. adopted a distinct approach by employing bacteriorhodopsin as a photoelectric transducer coupled with a specific peptide, establishing a novel photoelectric sensing platform for the detection of autoantibodies in rheumatoid arthritis; this facilitated efficient and accurate early screening (Contribution 5).

## 2. Applications for Food Safety, Agriculture, and Medical Diagnostics

Multiple studies in this Special Issue address concrete challenges in areas such as food safety, plant protection, and medical diagnostics. In the realm of food safety, Mei et al. developed a europium nanoparticle-based lateral flow strip biosensor for the rapid and simultaneous quantification of multiple prohibited quinoxalines in fish feed and tissues (Contribution 6). Duan et al. contributed to herbal medicine quality control by establishing a highly efficient ic-ELISA for the detection of gallic acid (Contribution 7). For plant virus prevention and control, Zhao et al. reported a highly specific immunochromatographic strip for cucumber green mottle mosaic virus, providing a practical tool for field monitoring (Contribution 8). Addressing global public health threats, Han et al. integrated ultrafast RT-PCR with *Pyrococcus furiosus* Argonaute-mediated nucleic acid detection into a one-tube platform capable of delivering results within 30 min, offering a promising alternative for rapid and accurate detection of SARS-CoV-2 and other RNA viruses (Contribution 9).

## 3. Emerging Interdisciplinary Technologies and Future Directions

This Special Issue also includes reviews and exploratory studies that highlight the deep integration of biosensing with other fields. The reviews by Liu et al. and Chen et al. systematically elaborate on the transformative potential of organ-on-chip technology in cancer research (Contribution 10) and the recent advances in microfluidic platforms for multiplex infectious disease detection, respectively, outlining a roadmap for the next generation of integrated, biomimetic, and automated biosensing technologies (Contribution 11). Several particularly noteworthy groundbreaking interdisciplinary studies are included. Faced with the challenge of generating antibodies to counter the mycotoxin patulin, Wang et al. innovatively applied four-dimensional data-independent acquisition quantitative proteomics to unravel the immune response mechanisms, providing valuable data and novel insights for optimizing antibody production against small-molecule toxins (Contribution 12). Oeyen et al. utilized cell-based electrical impedance biosensing to dynamically analyze virus–host interactions, identifying a novel role for CD300a in Zika virus entry and demonstrating the power of biosensing technologies in advancing fundamental virology research (Contribution 13).

## 4. Conclusions

This Special Issue presents a diverse and forward-looking collection of works that exemplify the innovation, versatility, and impact of modern immunoassays and biosensing technologies. Together, these contributions showcase how advances in molecular design, materials engineering, and analytical integration are transforming the landscape of biosensing, bridging fundamental research with practical applications in healthcare, food safety, environmental monitoring, and beyond.

We extend our sincere gratitude to all of the authors for their valuable contributions and to the reviewers and editors for their commitment to maintaining the scientific rigor and quality of this Special Issue.

